# Differential Expression of Serum TUG1, LINC00657, miR-9, and miR-106a in Diabetic Patients With and Without Ischemic Stroke

**DOI:** 10.3389/fmolb.2021.758742

**Published:** 2022-02-14

**Authors:** Omayma O Abdelaleem, Olfat G. Shaker, Mohamed M. Mohamed, Tarek I. Ahmed, Ahmed F. Elkhateeb, Noha K. Abdelghaffar, Naglaa A. Ahmed, Abeer A. Khalefa, Nada F. Hemeda, Rania H. Mahmoud

**Affiliations:** ^1^ Department of Medical Biochemistry and Molecular Biology, Faculty of Medicine, Fayoum University, Fayoum, Egypt; ^2^ Department of Medical Biochemistry and Molecular Biology, Faculty of Medicine, Cairo University, Giza, Egypt; ^3^ Department of Internal Medicine, Faculty of Medicine, Cairo University, Giza, Egypt; ^4^ Department of Internal Medicine, Faculty of Medicine, Fayoum University, Fayoum, Egypt; ^5^ Department of Critical Care, Faculty of Medicine, Fayoum University, Fayoum, Egypt; ^6^ Department of Clinical Pathology, Faculty of Medicine, Fayoum University, Fayoum, Egypt; ^7^ Department of Physiology, Faculty of Medicine, Zagazig University, Zagazig, Egypt; ^8^ Department of Genetics, Faculty of Agriculture, Fayoum University, Fayoum, Egypt

**Keywords:** TUG1, LINC00657, miR-9, miR-106a, stroke

## Abstract

**Background:** Ischemic stroke is one of the serious complications of diabetes. Non-coding RNAs are established as promising biomarkers for diabetes and its complications. The present research investigated the expression profiles of serum TUG1, LINC00657, miR-9, and miR-106a in diabetic patients with and without stroke.

**Methods:** A total of 75 diabetic patients without stroke, 77 patients with stroke, and 71 healthy controls were recruited in the current study. The serum expression levels of TUG1, LINC00657, miR-9, and miR-106a were assessed using quantitative real-time polymerase chain reaction assays.

**Results:** We observed significant high expression levels of LINC00657 and miR-9 in the serum of diabetic patients without stroke compared to control participants. At the same time, we found marked increases of serum TUG1, LINC00657, and miR-9 and a marked decrease of serum miR-106a in diabetic patients who had stroke relative to those without stroke. Also, we revealed positive correlations between each of TUG1, LINC00657, and miR-9 and the National Institutes of Health Stroke Scale (NIHSS). However, there was a negative correlation between miR-106a and NIHSS. Finally, we demonstrated a negative correlation between LINC00657 and miR-106a in diabetic patients with stroke.

**Conclusion:** Serum non-coding RNAs, TUG1, LINC00657, miR-9, and miR-106a displayed potential as novel molecular biomarkers for diabetes complicated with stroke, suggesting that they might be new therapeutic targets for the treatment of diabetic patients with stroke.

## Introduction

Diabetes mellitus (DM) is a complex, multisystem disease and is one of the risk factors of stroke. Oxidative stress occurs due to an elevated blood glucose level, which is associated with increased glycated end products, resulting in endothelial dysfunction, cerebrovascular atherosclerosis, and thrombosis, which are the main causes of ischemic stroke in diabetic patients that is associated with high mortality and poor prognosis (Nakagami et al., 2005; Ferreiro et al., 2010).

It is important to understand the molecular mechanisms of cerebral stoke associated with DM to facilitate the development of new effective potential biomarkers and therapeutic targets for diabetic patients with stroke.

Non-coding RNAs, including microRNAs (miRNAs) and long non-coding RNAs (lncRNAs), have been proven to have necessary roles in regulating gene expression (Kitagawa et al., 2013; Iyengar et al., 2014; Khoshnam et al., 2017). LncRNAs are an important group of non-coding RNAs that are of long transcripts (>200 bp). Numerous studies have elucidated the significant roles of lncRNAs in various diseases, including ischemic stroke (Mercer and Mattick, 2013; Bao et al., 2018a).

Taurine‐upregulated gene 1 (TUG1), a lncRNA, has been shown to be related to the pathogenesis of many diseases. Recently, TUG1 has gained significant attention in ischemic injuries (Long et al., 2016; Chen et al., 2017; Wang et al., 2017), although little has been identified regarding its role in DM complicated with stroke.

LINC00657 is a lncRNA that is highly conserved and profusely expressed in endothelial cells (Michalik et al., 2014). Accumulating evidence has demonstrated that LINC00657 might play an oncogenic role, and it is upregulated in many cancers (Liu H. et al., 2016; Liu S. et al., 2016). However, its role in DM or diabetes-related complications has not been investigated yet.

MicroRNAs (miRNAs) are tiny non-coding RNAs (20–25 nucleotides long). Recently, promising research studies have explained the importance of miRNAs in the pathogenesis of diabetes and its cardiovascular complications (Meng et al., 2012; Koutsis et al., 2013). However, the expressions of miR-9 and miR-106a in diabetic patients with ischemic stroke have not been examined.

Bioinformatics has reported that TUG1 has complementary sequences of miR-9. Additionally, LINC00657 contains binding sites for miR-106a (Li et al., 2014). However, a study of their relationship in DM complicated with cerebral stroke remains to be conducted.

In this study, we aimed to assess the serum expression levels of TUG1, LINC00657, miR-9, and miR-106a in diabetic patients who had stroke and those without cerebral stroke and to explore any association between these non-coding RNAs and clinico-laboratory data.

### Subjects and Methods

#### Study Population

**FIGURE 1:**
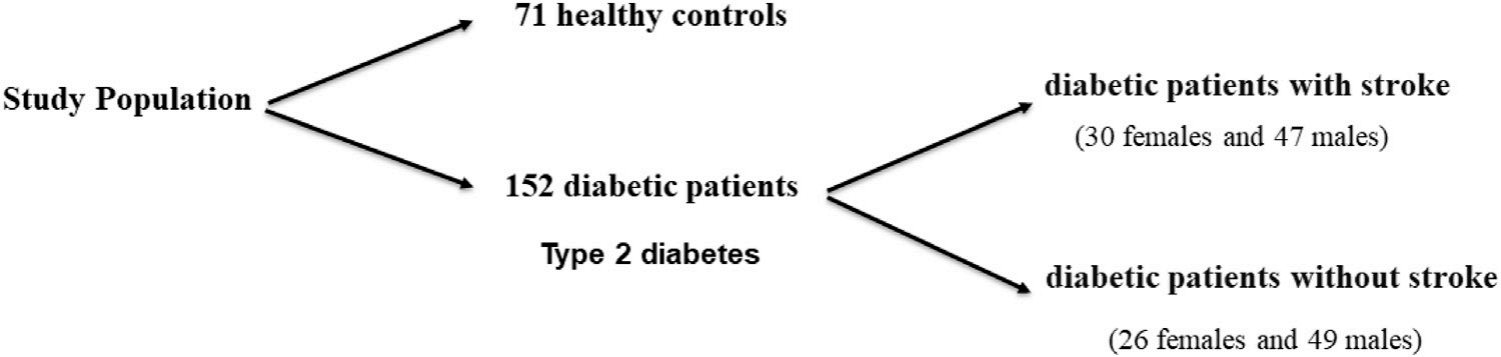
Schematic diagram of the outline of the work performed in this study.

A total of 152 diabetic patients (with type 2 diabetes) were recruited among those admitted to the outpatient and inpatient clinics of the Internal Medicine and Intensive Care Unit, Fayoum University Hospital, Fayoum, in the period from November 2019 to December 2020. Diabetic patients were selected based on the American Diabetes Association 2015 diagnostic criteria (Pinsker et al., 2015). Diabetic patients were divided into two groups: diabetic patients with stroke (30females and 47males, with a mean = 57.08 ± 16.31 years) and diabetic patients without stroke (26 females and 49 males, mean age = 53.19 ± 17.78 years) (Figure 1 **)**.

Ischemic stroke diagnosis was assessed according to clinical symptoms and physical examinations, and this diagnosis was confirmed by computed tomography (CT) or magnetic resonance imaging (MRI). The National Institutes of Health Stroke Scale (NIHSS) was used by experienced neurologists to evaluate the neurological deficits.

All patients with brain tumors, intracerebral hemorrhage, recurrent stroke, history of hypertension, recent head injuries, immune system disorders, liver or renal diseases, blood diseases, acute infectious diseases, or a family history of stroke were excluded from the study. Furthermore, 71 healthy individuals (age and sex matched to the patients) who did not have systemic or neurologic diseases were considered as the control group in this study.

Written informed consent was signed by all enrolled participants after a detailed explanation of the study. The study protocol was performed in agreement with the Declaration of Helsinki. The Ethics Committee of the Faculty of Medicine, Fayoum University, approved this research protocol.

### Serum Collection

From each participant, 5 ml of venous blood was collected into plain tubes using a Vacutainer system following a 12-h fast. Serum separator tubes were used to collect the samples that were left for 15 min to clot. Centrifugation at 4,000 × *g* for 10 min was performed, separating the serum that was stored at −80°C until the time of use. An extra blood sample was taken 2 h after a meal (2 h post-prandial, 2hPP) into tubes containing fluoride.

Fasting blood glucose (FBG), 2hPP blood glucose, cholesterol, triglycerides, HbA1C, creatinine, and low-density lipoprotein (LDL) were assessed using standard methods on cobas c311 (Roche, Mannheim, Germany) in accordance with the instructions in the kit. Serum samples were used for the quantification of TUG1, LINC00657, miR-9, and miR-106a using real-time PCR.

### LncRNA and miRNA Extraction and Reverse Transcription

According to the manufacturer’s protocol, total RNA (including lncRNAs and miRNAs) was extracted from serum samples using the miRNeasy extraction kit (Qiagen, Hilden, Germany) after adding the QIAzol lysis reagent. Quantitation and the purity of the RNA samples were assessed using the NanoDrop^®^ (ND)-1000 spectrophotometer (NanoDrop Technologies, Inc., Wilmington, DE, USA).

Complementary DNA (cDNA) was generated from the extracted RNA in a total volume of 20 μl/reaction using the RT^2^ First Strand Kit (Qiagen, Germantown, MD, USA) according to the manufacturer’s protocol for lncRNA expression analysis. Moreover, the miScript II RT Kit (Qiagen, Valencia, CA, US) was used for miRNA expression analysis in a 20-μl reverse transcription (RT) reaction according to the instructions in the pamphlet.

### LncRNA and miRNA Expression by Real-Time Quantitative PCR

Real-time PCR amplification reactions were performed using the RT^2^ SYBR Green PCR Kit (Qiagen, Germantown, MD, USA) for the detection of lncRNA. However, the miScript SYBR Green PCR Kit (Qiagen, Valencia, CA, USA) was used in the quantification of miRNAs with the aid of the Rotor-Gene Q System (Qiagen).

The RefSeq accession no. of TUG1 was NR_002323.2 and that of LINC00657was NR_027451.1. GAPDH was used as an endogenous control for the evaluation of TUG1 and LINC00657 according to the manufacturer’s instructions. Numerous studies have used GAPDH as an internal reference for serum lncRNAs (Duan et al., 2016; Shaker et al., 2019). The primer sequences of GAPDH were as follows: forward: 5′-CCC​TTC​ATT​GAC​CTC​AAC​TA-3′; reverse: 5-′TGG​AAG​ATG​GTG​ATG​GGA​TT-3′.

Moreover, the catalog number of miR-9 was MS00010752 and that of miR-106a was MS00008393. SNORD68 was used as the internal reference for the evaluation of the gene expression levels of miR-9 and miR-106a. The catalog number of *SNORD68* was MS00033712.

The PCR cycling program for the quantification of lncRNAs consists of an initial incubation at 95°C for 10 min, followed by 40 cycles at 95°C for 15 s and 60°C for 60 s. That for the detection of miRNAs consists of 95°C for 30 min, followed by 40 cycles at 94°C for 15 s, 55°C for 30 s, and 70°C for 30 s.

The relative expression levels of TUG1, LINC00657, miR-9, and miR-106a were calculated using 2^−ΔΔCt^. Fold change (FC) values less than 1 indicated downregulation, while values more than 1 indicated upregulation of non-coding RNAs (Livak and Schmittgen, 2001). Control FC values were set as 1.

### Statistical Analyses

Statistical analysis was performed using Statistical Package for Social Sciences (SPSS) version 24. The mean, standard deviation (SD), median, and interquartile range (IQR) were utilized to represent quantitative data. A chi-square test was performed for categorical data. However, the Mann–Whitney *U* test was used for continuous variables, which were presented as median (interquartile range). To determine the relation of the expressions of non-coding RNAs with the study parameters, Spearman’s correlation was run. A multivariate stepwise logistic regression was constructed to identify the significant predictors of cerebral stroke among the four markers.

Analyses of the receiver operating characteristic (ROC) curves were conducted to determine the sensitivity and specificity of TUG1, LINC00657, miR-9, and miR-106a as predictors in differentiating between different groups. Statistical significance was considered at a *p*-value <0.05. Adjusted *p*-values for multiple comparisons of the studied groups were estimated using the Bonferroni correction method. The *p*-value (of 0.05) was divided by the number of comparisons, i.e., 3 (0.05/3). Therefore, the test results were considered to be statistically significant at *p*-values <0.017.

## Results

### Clinical and Laboratory Features of the Enrolled Participants

A total of 75 diabetic patients without stroke, 77 diabetic patients with stroke, and 71 healthy individuals were included in the current study.

There were marked differences between the total diabetic patients (with and without stroke) and the healthy group regarding FBG, 2hPP, HbAIc, alanine transaminase (ALT), aspartate transaminase (AST), urea, creatinine, cholesterol, LDL, high-density lipoprotein (HDL), and triglycerides (all *p* < 0.05). However, no significant differences in age, sex, and other clinical and laboratory data were observed between all diabetic patients and control participants (*p* > 0.05) (Table 1). Moreover, there were significant differences concerning FBG, 2hPP, HbAIc, and LDL when comparing diabetic patients with stroke to those without stroke (all *p* < 0.05). On the other hand, there were no marked differences regarding age, sex, and all other data when comparing diabetic patients with stroke to those without stroke (*p* > 0.05) (Table 1).

**TABLE 1:** Baseline characteristics of the enrolled groups

	**Control (*n* = 71)**	**Diabetes**		***p*-value** ^**a**^	***p*-value** ^**b**^
		**Without stroke (*n* = 75)**	**With stroke (*n* = 77)**		
Sex, *n* (%)					
Female	25 (35.21%)	26 (34.67%)	30 (38.96%)	0.441	0.352
Male	56 (64.79%)	49 (65.33%)	47 (61.04%)	0.553	0.907
Age (years)	54.58 ± 18.75	53.19 ± 17.78	57.08 ± 16.31	0.894	0.559
BMI (kg/m^2^)	29.37 ± 1.82	30.07 ± 2.46	31.89 ± 2.09	0.389	0.604
FBG (mg/dl)	83.25 ± 8.97	154.58 ± 28.11	185.41 ± 40.85	<0.001*	0.04*
2hPP (mg/dl)	111.85 ± 10.24	255.75 ± 47.08	309.15 ± 53.29	<0.001*	0.04*
HbA1c (%)	4.27 ± 1.65	7.87 ± 2.34	9.07 ± 3.12	<0.001*	0.02*
ALT (IU/L)	18.74 ± 3.89	37.25 ± 9.27	40.09 ± 8.97	<0.001*	0.07
AST (IU/L)	17.92 ± 8.17	32.25 ± 6.87	35.51 ± 8.71	0.002*	0.425
Urea (mg/dl)	24.71 ± 8.74	55.19 ± 11.31	58.18 ± 9.47	<0.001*	0.108
Creatinine (mg/dl)	0.70 ± 0.19	2.72 ± 0.34	3.09 ± 0.17	0.02*	0.094
Hb (gm/dl)	12.13 ± 3.24	11.89 ± 2.98	12.01 ± 3.07	0.498	0.571
MCV	33.12 ± 2.19	33.09 ± 2.01	32.97 ± 2.05	0.608	0.580
MCH	28.11 ± 2.31	27.98 ± 3.07	29.01 ± 2.13	0.333	0.231
Cholesterol (mg/dl)	138.15 ± 23.19	168.25 ± 18.52	198.16 ± 34.08	0.008*	0.06
LDL (mg/dl)	49.57 ± 19.87	86.17 ± 15.79	101.71 ± 25.55	<0.001*	0.03*
HDL (mg/dl)	41.22 ± 8.99	35.12 ± 7.58	30.09 ± 8.88	0.03*	0.064
Triglycerides (mg/dl)	65.13 ± 9.13	137.32 ± 35.62	149.85 ± 44.73	<0.001*	0.091
NIHSS	–	–	11.38 ± 5.12	–	–
Disease duration (years)	–	13.35 ± 1.87	15.729 ± 1.97	–	0.09

Data are shown as the mean ± ( SD, median (range), or *n* (%).

*BMI*, body mass index; *FBG*, fasting blood glucose; *2hPP*, 2 h post-prandial; *HbA1c*, glycated hemoglobin A1c; *ALT*, alanine transaminase; *AST*, aspartate transaminase; *Hb*, hemoglobin; *MCV*, mean corpuscular volume; *MCH*, mean corpuscular hemoglobin; *LDL*, low-density lipoprotein; *HDL*, high-density lipoprotein; *NIHSS*, National Institutes of Health Stroke Scale

*Significant at *p* < 0.05

a Comparison of diabetic patients (with and without stroke) *versus* the healthy control group

b Comparison of diabetic patients with stroke *versus* diabetic patients without stroke

### Comparison of the Serum Expression Levels of TUG1, LINC00657, miR-9, and miR-106a in the Different Studied Groups

As clarified in Table 2, the serum expression levels of LINC00657 and miR-9 were increased significantly in diabetic patients without stroke when compared to healthy individuals (*p* = 0.001 for LINC00657 and miR-9).

**TABLE 2:** Expression levels of serum TUG1, LINC00657, miR-9, and miR-106a in all groups

**Variables**	**Diabetes without stroke (*n* = 75)**	**Diabetes with stroke (*n* = 77)**	***p*-value**
	**Median (intraquartile range)**		
TUG1	0.71 (0.01–1.97)	2.90 (0.31–13.25)	0.125^a^ < 0.001*^b^ ^,^ ^c^
LINC00657	3.18 (0.87–20.98)	11.85 (0.50–53.85)	0.001*^a^ < 0.001*^b^ ^,^ ^c^
miR-9	1.45 (0.12–8.07)	4.40 (0.35–12.25)	0.001*^a^ < 0.001*^b^ 0.003*^c^
miR-106a	0.760 (0.13–1.62)	0.03 (0.01–0.41)	0.09^a^ < 0.001*^b^0.05^c^

Fold change levels represent non-coding RNA expression relative to controls that were calculated using 2^−ΔΔCT^. Control fold change levels are equivalent to 1. Data are expressed as the median and intraquartile range. Adjusted *p*-values for multiple comparisons of the studied groups were estimated using the Bonferroni correction method.

*Significant at *p* < 0.017

a Comparison of diabetes without stroke *versus* healthy controls

b Comparison of diabetes with stroke *versus* healthy controls

c Comparison of diabetes with stroke *versus* diabetes without stroke

We next compared the expressions of TUG1, LINC00657, miR-9, and miR-106a in the sera of diabetic patients with stroke relative to healthy controls. The results showed significant upregulation of TUG1, LINC00657, and miR-9 (*p* < 0.001 for TUG1, LINC00657, and miR-9). In contrast, the level of miR-106a in serum was markedly decreased in diabetic patients who had stroke relative to the control subjects (*p* < 0.001).

Furthermore, we revealed a marked elevation of the serum expressions of TUG1, LINC00657, and miR-9 in diabetic patients with stroke relative to those without stroke (*p* < 0.001 for TUG1 and LINC00657; *p* = 0.003 for miR-9). Meanwhile, a non-significant decrease of miR-106a was detected between diabetic patients with stroke and those without stroke (*p* = 0.05).

### Correlation of TUG1, LINC00657, miR-9, and miR-106a With Stroke Severity and Clinical Characteristics

NIHSS scoring was performed to evaluate stroke severity. We used Spearman’s analysis to assess the correlation between the aforementioned ncRNAs and stroke severity, as well as clinical and laboratory data.

As demonstrated in Table 3, TUG1, LINC00657, and miR-9 were positively correlated with NIHSS (*r* = 0.802, *p* < 0.001; *r* = 0.709, *p* < 0.001; and *r* = 0.681, *p* < 0.001, respectively). At the same time, a negative correlation was shown between miR-106a and NIHSS (*r* = −0.569, *p* = 0.001). In addition, TUG1, LINC00657, and miR-9 were positively correlated with disease duration (*r* = 0.754, *p* < 0.001; *r* = 0.720, *p* < 0.001; and *r* = 0.675, *p* < 0.001, respectively). On the other hand, a negative correlation was observed between miR-106a and years since the occurrence of DM (*r* = −0.600, *p* < 0.001).

**TABLE 3:** Correlation between the expression levels of serum non-coding RNAs and clinical parameters in diabetic patients with stroke

**Variables**	**TUG1**	**LINC00657**	**miR-9**	**miR-106a**
Disease duration	0.754 (<0.001)*	0.720 (<0.001)*	0.675 (<0.001)*	−0.600 (<0.001)*
NIHSS	0.802 (<0.001)*	0.709 (<0.001)*	0.681 (<0.001)*	−0.569 (0.001)*
Age	0.097 (0.821)	**−**0.074 (0.893)	0.107 (0.275)	**−**0.197 (0.104)
BMI	0.099 (0.752)	0.055 (0.708)	0.122 (0.564)	**−**0.189 (0.262)
FBG	0.017 (0.920)	0.213 (0.206)	0.109 (0.523)	0.269 (0.107)
2hPP	0.094 (0.578)	0.172 (0.310)	0.102 (0.548)	0.114 (0.501)
HbA1c	−0.014 (0.779)	0.098 (0.587)	0.073 (0.669)	−0.107 (0.527)
AST	0.034 (0.839)	0.098 (0.565)	0.013 (0.941)	0.277 (0.096)
ALT	−0.123 (0.467)	−0.187 (0.267)	−0.085 (0.618)	−0.264 (0.114)
Urea	−0.076 (0.653)	−0.146 (0.388)	−0.123 (0.467)	−0.138 (0.416)
Creatinine	−0.127 (0.454)	−0.115 (0.499)	−0.125 (0.460)	0.069 (0.686)
Cholesterol	0.101 (0.552)	0.136 (0.421)	0.187 (0.268)	−0.131 (0.441)
LDL	0.037 (0.830)	−0.053 (0.755)	−0.052 (0.684)	0.171 (0.244)
HDL	−0.012 (0.890)	0.113 (0.474)	0.124 (0.466)	−0.165 (0.328)
Triglycerides	0.051 (0.765)	0.011 (0.872)	0.029 (0.865)	−0.097 (0.524)

*BMI*, body mass index; *FBG*, fasting blood glucose; *2hPP*, 2 h post-prandial; *HbA1c*, glycated hemoglobin A1c; *ALT*, alanine transaminase; *AST*, aspartate transaminase; *Hb*, hemoglobin; *LDL*, low-density lipoprotein; *HDL*, high-density lipoprotein; *NIHSS*, National Institutes of Health Stroke Scale

*Significant at *p* < 0.05

However, no significant correlation was detected between TUG1, LINC00657, miR-9, and miR-106a and the laboratory parameters in the present study (all *p* > 0.05).

### Correlation of TUG1 With miR-9 and of LINC00657 With miR-106a

Interestingly, the current results reported a negative correlation between LINC00657 and miR-106a (*r* = −0.507, *p* = 0.002) in diabetic patients with stroke. However, no significant correlation was shown between the serum levels of TUG1 and miR-9 (*r* = 0.251, *p* = 0.10).

### ROC Analysis to Determine the Diagnostic Performance of Serum TUG1, LINC00657, miR-9, and miR-106a in Distinguishing Diabetic Patients With Stroke From Control Subjects

**FIGURE 2:**
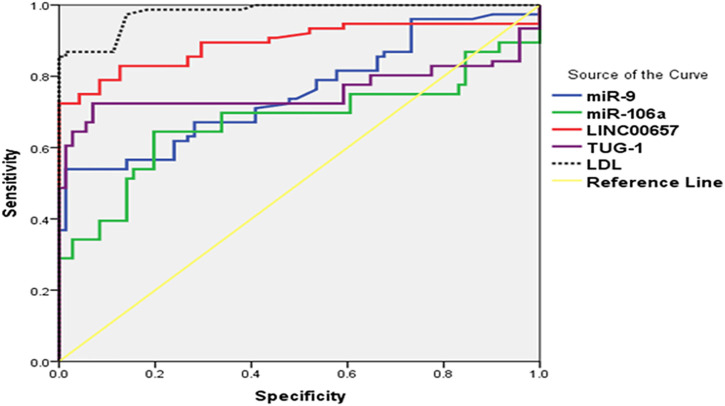
(ROC) curve analysis of serum TUG1, LINC00657, miR-9, and miR-106a for distinguishing diabetic patients without stroke from control subjects.

An ROC curve was assembled to estimate the diagnostic value of TUG1, LINC00657, miR-9, and miR-106a as novel biomarkers for DM with stroke relative to healthy subjects. For TUG1, the AUC was 0.758 (95% CI = 0.669–0.846, *p* < 0.001), with a sensitivity of 48.50% and a specificity of 100%. Moreover, the AUC of LINC00657 was 0.892 (95% CI = 0.834–0.950, *p* < 0.001), with a sensitivity of 73.50% and a specificity of 100%. Also, the AUC of miR-9 was 0.755 (95% CI = 0.677–0.834, *p* < 0.001), with a sensitivity of 39.5% and a specificity of 100%. Regarding miR-106a, its AUC was 0.674 (95% CI = 0.583–0.765, *p* < 0.001), and the sensitivity and specificity were 38.4% and 100%, respectively. On the other hand, the AUC of LDL was 0.979 (95% CI = 0.962–0.996, *p* < 0.001), with sensitivity of 87.5% and specificity of 45.8% (Table 4 and Figure 2).

**TABLE 4:** Receiver operating characteristics (ROC) curve analysis using serum TUG1, LINC00657, miR-9, miR-106a, and LDL for discriminating diabetic patients with stroke from control subjects

**Variable**	**AUC (95% CI)**	***p*-value**	**Sensitivity (%)**	**Specificity (%)**	**Total accuracy**
TUG1	0.758 (0.669–0.846)	<0.001*	48.50	100	74.25
LINC00657	0.892 (0.834–0.950)	<0.001*	73.50	100	86.75
miR-9	0.755 (0.677–0.834)	<0.001*	39.5	100	69.75
miR-106a	0.674 (0.583–0.765)	<0.001*	38.4	100	69.20
LDL	0.979 (0.962–0.996)	<0.001*	87.5	45.8	66.65

*AUC*, area under the curve; *CI*, confidence interval; *LDL*, low-density lipoprotein

*Significant at *p* < 0.05.

ROC curve analysis revealed that serum TUG1, LINC00657, miR-9, and miR-106a have good value as prognostic markers in discriminating diabetic patients with stroke from those without stroke.

**FIGURE 3:**
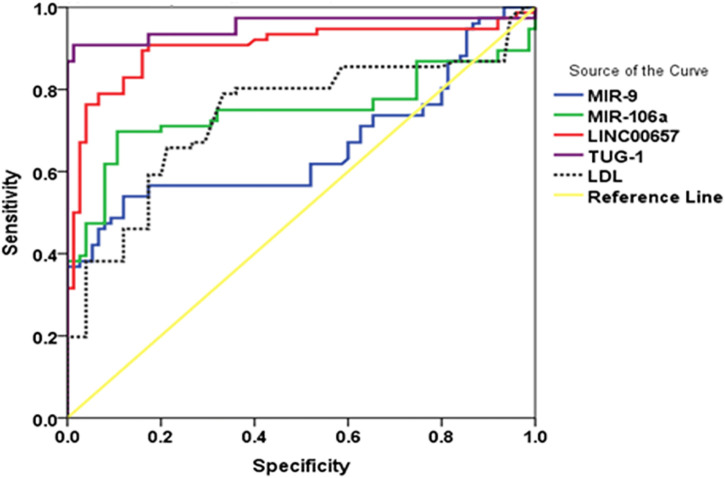
(ROC) curve analysis of serum TUG1, LINC00657, miR-9, and miR-106a for discriminating diabetic patients with stroke from those without stroke.

The current results demonstrated that using TUG1 to diagnose diabetes with stroke yielded an AUC of 0.954 (95% CI = 0.915–0.994, *p* < 0.001), with a sensitivity of 87.9% and a specificity of 98.5%. In addition, the AUC value for LINC00657 was 0.902 (95% CI = 0.847–0.957, *p* < 0.001), with a sensitivity of 35.1% and a specificity of 98.5%. Also, miR-9 had an AUC of 0.661 (95% CI = 0.571–0.752, *p* < 0.001) and sensitivity and specificity values of 39.0% and 93.5%, respectively. For miR-106a, the AUC was 0.747 (95% CI = 0.661–0.832, *p* = 0.01), with a sensitivity of 39.0% and a specificity of 100%, while the AUC of LDL was 0.736 (95% CI = 0.654–0.819, *p* < 0.001) and the sensitivity and specificity were 20% and 90.2%, respectively (Table 5 and Figure 3).

**TABLE 5:** Receiver operating characteristics (ROC) curve analysis using serum TUG1, LINC00657, miR-9, and miR-106a for discriminating diabetic patients with stroke from diabetic patients without stroke

**Variable**	**AUC (95% CI)**	***p*-value**	**Sensitivity (%)**	**Specificity (%)**	**Total accuracy**
TUG1	0.954 (0.915–0.994)	<0.001*	87.9	98.5	93.2
LINC00657	0.902 (0.847–0.957)	<0.001*	35.1	98.5	66.8
miR-9	0.661 (0.571–0.752)	0.001*	39.0	93.5	51.25
miR-106a	0.747 (0.661–0.832)	<0.001*	39.0	100	69.5
LDL	0.736 (0.654–0.819)	<0.001*	20	90.2	55.1

*AUC*, area under the curve; *CI*, confidence interval; *LDL*, low-density lipoprotein

*Significant at *p* < 0.05.

### Multiple Logistic Regression Analysis

Multivariate regression analysis (considering NIHSS as the dependent variable) confirmed that TUG1 and LINC00657 were independent predictors for diabetes with stroke (*p* = 0.04 and *p* = 0.01, respectively) (Table 6).

**TABLE 6:** Multiple logistic regression analysis

	***B***	**SE**	***p-*value**	**95% CI for *B***	
				**Lower**	**Upper**
TUG1	−5.980	1.071	0.02*	2.82	11.2
LINC00657	−19.248	2.09	0.03*	1.02	5.54
miR-9	−0.633	0.115	0.124	0.91	2.11
miR-106a	4.135	3.294	0.247	0.44	5.52
LDL	0.012	0.007	0.078	−0.001	0.025
Disease duration	0.243	0.082	0.004*	0.080	0.406
FBG	−0.005	0.017	0.786	−0.038	0.029
HbA1c	−0.311	−0.196	0.117	−0.702	0.080
Constant	9.464	3.688	0.013	2.100	16.828

*CI*, confidence interval; *LDL*, low-density lipoprotein; *FBG*, fasting blood glucose; *2hPP*, 2 h post-prandial; *HbA1c*, glycated hemoglobin A1c

*Significant at *p* < 0.05

## Discussion

Type 2 diabetes mellitus (T2DM) has emerged as a cause of serious concern worldwide and has been established as a risk factor for ischemic stroke (Nakagami et al., 2005). It is important to discover new sensitive and easily detected biomarkers for the diagnosis and prognosis of T2DM and its related complications.

Recently, it has been shown that non-coding RNAs (including lncRNAs and microRNAs) may be used as probable biomarkers for T2DM and its associated complications due to their stability and differential expression in a variety of body fluids, such as plasma and serum (Mastropasqua et al., 2014; Shaker et al., 2019). However, no previous reports have investigated the role of TUG1, LINC00657, miR-9, and miR-106a in stroke associated with DM. Thus, in this article, we assessed the serum expression levels of TUG1, LINC00657, miR-9, and miR-106a in diabetic patients with and without stroke.

We observed low TUG1 and miR-106a and significantly high LINC00657 and miR-9 expression levels in the serum of diabetic patients without stroke compared to control participants. At the same time, we verified marked increases of serum TUG1, LINC00657, and miR-9 and a marked decrease of serum miR-106a in diabetic patients who had stroke relative to those without stroke. Previous studies reported that the expression of TUG1 was decreased in rats with diabetes and in mesangial cells induced with high-level glucose through inhibition of the PI3K/AKT pathway (Zang et al., 2019). Furthermore, Wang et al. showed that TUG1 was downregulated in NRK-52E cells (high-glucose-stimulated) in mice *via* targeting miR-21 (Wang et al., 2019). Similarly, Li et al. documented a low expression level of TUG1 in high-glucose-stimulated podocytes by hindering the expression of miR-27a-3p (Li et al., 2019).

Our results regarding the upregulation of TUG1 in diabetic patients who had stroke are in line with recent studies showing that TUG1 was overexpressed in ischemic stroke by regulating miR-9 and decreasing Bcl-2-like 11 protein [25]. Also, in atherosclerosis, the elevated expression level of TUG1 increased endothelial cell apoptosis through miR-26a sponging (Chen et al., 2016). Similarly, many recent studies have discussed the role of TUG1 in atherosclerosis. For example, Li et al. found that TUG1*, via* regulating the miR-21/PTEN axis, increased the proliferation of vascular smooth muscle (Li et al., 2018). In addition, Yan et al. documented the role of TUG1 in the migration and proliferation of endothelial cells by the Wnt pathway (Yan et al., 2018). Moreover, Zhang et al. reported that TUG1 knockdown ameliorated atherosclerotic lesion and inhibited inflammation and hyperlipidemia *via* the upregulation of fibroblast growth factor 1 (Zhang et al., 2018). Besides, Yang et al. noted an increased expression level of TUG1 in ischemic heart exposed to oxidative stress *via* increasing cardiomyocyte apoptosis (Yang et al., 2019). However, there are no reports on the relation between TUG1 and stoke associated with DM.

Regarding LINC00657 (NORAD), our results are in line with a study which found that LINC00657, which is expressed in vascular endothelial cells, induced angiogenesis during atherosclerosis through the upregulation of VEGF, MMP-2, and MMP-9 (Wan et al., 2020). Also, Michalik et al. revealed that LINC00657 was markedly elevated during hypoxia (Michalik et al., 2014). Of note is that Bao et al. reported that oxidized LDL (oxLDL) treatment, which promotes oxidative stress and is implicated in atherosclerosis, resulted in the overexpression of LINC00657 (Bao et al., 2018b). Since hypoxia and atherosclerosis are predisposing factors of ischemic stroke, we therefore assumed that LINC00657 might contribute to the pathogenesis of stroke.

In the current research, we assessed the expression level of miR-9, which is a target gene of TUG1 and miR-106a, which are target genes of LINC00657. It was revealed in previous studies that the serum expression level of miR-9 increased significantly in T2DM (Kong et al., 2011), which is in accordance with our results. Furthermore, miR-9 was found to decrease insulin secretion *via* targeting syntaxin-binding protein 1, Onecut 2, and sirtuin 1 (Sirt1) (Plaisance et al., 2006; Ramachandran et al., 2011; Hu et al., 2018). However, Jiménez-Lucena et al. reported a low plasma level of miR-9 in patients at risk of T2DM (Jiménez-Lucena et al., 2018).

More importantly, previous studies also demonstrated the role of miR-9 in ischemic stroke, such as Ji et al. who found that miR-9 was upregulated in the serum exosomes of patients with acute ischemic stroke and was strongly associated with interleukin 6 (IL-6) production (Ji et al., 2016). In addition, the serum expression level of miR-9 was verified to be elevated significantly in acute ischemic stroke patients and was positively correlated with inflammatory markers, infarct volume, and the NIHSS score (Ji et al., 2016). Besides, another study has considered miR-9 to be a new biomarker of neurotoxicity and neural damage (Xue et al., 2018). At the same time, an increasing number of studies have explained the role of miR-9 in neuronal apoptosis after ischemic stroke (Wei et al., 2016).

Concerning miR-106a, our findings are in line with the study by Wu et al., which determined that miR-106a was decreased in diabetic peripheral neuropathy *via* the regulation of 12/15-lipoxygenase of oxidative/nitrative stress (Wu et al., 2017). Previous findings demonstrated the role of miR-106a in numerous risk factors of ischemic stroke. For example, under oxidative stress, miR-106-5p was documented to be decreased, causing premature senescence by suppressing the G1/S-phase transition of the cell cycle through modulating the expression of E2F1 (Tai et al., 2020). Similarly, increased levels of reactive oxygen species (ROS) resulted to the decreased expression of miR-106a (Wang et al., 2010). On the other hand, elevated levels of miR-106a prevented oxidative stress injury and inflammation in hepatic mouse with gestational hypertension (Wang Z. et al., 2019), resulting to repression of the expressions of HIF1-α and VEGF in diabetic retina (Ling et al., 2013). In addition, miR-106a has been associated with macrophage activation, suggesting its involvement in inflammation (Zhu et al., 2013).

In the present work, it was interesting to find a negative correlation between LINC00657 and miR-106a in diabetic patients who had stroke. A number of recent studies have hypothesized that lncRNAs could affect the progression of diseases through regulating miRNAs. It was reported that LINC00657 could influence tumorigenesis in hepatocellular carcinoma by regulating miR-106a (Hu et al., 2017).

Notably, an ROC curve was constructed in our study. The results implied that serum TUG1, LINC00657, miR-9, and miR-106a could discriminate diabetic patients without stroke from healthy subjects. More importantly, the aforementioned non-coding RNAs may be used to differentiate diabetic patients with stroke from those without stroke.

Some limitations of this work should be addressed. First is the relatively small sample size. Therefore, further works with larger sample sizes in various populations are needed. Moreover, further experiments are necessary to explain the detailed mechanisms of the role of these non-coding RNAs in diabetic patients with and without stroke.

## Conclusion

The current study, for the first time, revealed that serum TUG1, LINC00657, miR-9, and miR-106a may serve as novel potential indicators of stroke associated with diabetes and correlated significantly with NIHSS. Furthermore, they might be used as new targets of treatment for diabetic patients with stroke.

## Data Availability

The datasets presented in this article are not readily available due to patient confidentiality. Requests to access the datasets should be directed to dr.omayma@yahoo.com.
